# Mindfulness and Coaching to Improve Learning Abilities in University Students: A Pilot Study

**DOI:** 10.3390/ijerph17061935

**Published:** 2020-03-16

**Authors:** Lorenza Corti, Carmen Gelati

**Affiliations:** 1 Department of Electrical Engineering and Information Technologies, University of Naples Federico II, 80125 Naples, Italy; 2 Department of Psychology, University of Milano-Bicocca, 20126 Milan, Italy; carmen.gelati@unimib.it

**Keywords:** self-regulated learning, self-regulated study, academic emotions, study motivation, effective learning, mindfulness, mindfulness-based coaching, study strategies, study techniques, anxiety for studying

## Abstract

This pilot study investigated the effects of a short 10-module intervention called MEL (Mindful Effective Learning), which integrates mindfulness, coaching, and training on study strategies, to improve learning abilities among university students. Inspired by ample research on the learning topics that points out how effective learning and good academic results depend simultaneously on self-regulation while studying combined with emotional and motivational factors, the intervention aimed to train students simultaneously in these three aspects. The intervention group participants (*N* = 21) and the control group participants (*N* = 24) were surveyed pre- and post-intervention with the Italian questionnaire AMOS (Abilities and Motivation to Study) and the Italian version of the Mindful Attention Awareness Scale (MAAS). The results showed that, regarding self-regulation in study, trained students improved their self-awareness, self-evaluation ability, metacognition skills, and organizational and elaborative ability to manage study materials; regarding emotional aspects, they improved their anxiety control; regarding motivation they developed an incremental theory of Self and improved their confidence in their own intelligence. Moreover, two follow-up self-report surveys were conducted, and trained students reported positive assessments of the MEL intervention. Findings suggest that a short intervention based on mindfulness and coaching and training on study strategies may improve students’ effective learning.

## 1. Introduction

Research has shown that effective learning requires metacognitive and affective skills in addition to cognitive and academic knowledge and skills. These metacognitive and affective factors include: self-regulation abilities in study [1,2,3] emotions [4,5,6] and motivation in study and academic achievement goals [7,8]. It has also been found that these three dimensions are linked to each other and are often overlapped and that it is useful to take all of them into account [2,9,10].

### 1.1. Self-Regulated Learning, Emotion Regulation, and Achievement Motivation

Effective study at the college level requires self-regulated learning (SRL) that occurs when learners have an active role in their learning and are aware of how they regulate their study [1,2,3,11]. Self-regulated learning includes the ability to effectively use study strategies and techniques such as organization of study activities, elaborating study content, monitoring and evaluating one’s studying, and use of retention techniques [12,13,14,15], and on metacognition skill, which is the ability to monitor the functioning of one’s mind [16,17,18,19]. In a recent paper, Panadero [2] reviewed different models which describe self-regulated learning. He stated that self-regulated learning is a big umbrella that includes emotional and motivational aspects.

Emotions play an important role in learning and in academic achievement, as stated by Pekrun’s control-value theory [4,5]. This theory defines academic achievement emotions as emotions directly related to the achievement activities of studying. It is important for students to acquire abilities to regulate them. Concerning academic emotional regulation abilities, among all the academic emotions, anxiety is important to investigate because, if not regulated, it can be a great obstacle to success in learning and achieving one’s academic goals [6].

Furthermore, motivation is a crucial factor for effective learning and academic goal achievement [3,8,11]. Motivation is a multidimensional construct that can be described by using several important contributions [9], such as the Dweck’s Self-theories [20], the concept of self-efficacy introduced by Bandura [21] and achievement goal theory [22]. The social-cognitive approach to motivation, Dweck’s Self-theories [20], deals with beliefs and argues that students with an incremental theory of their own intelligence and personality have more academic success compared to students with a fixed vision. Concerning the second contribution, research has highlighted that students who experience high self-efficacy have greater potential for academic success [7,23,24]. Finally, achievement goal theory shows that students can have mastery or performance achievement goals related to an incremental or fixed perception of Self, respectively. Therefore, it is important to foster mastery more than performance goals in students [22]. Being able to manage one’s own learning also means facing failure and maintaining one’s motivation and desire for success. For this reason, resilience, that is coping ability, can be taken into account; resilience is a transversal ability related to self-regulation in study, emotions and motivation, and it plays an important role in the achievement of academic results [25,26]. 

To train students to pursue effective learning and academic achievements, research has shown that both mindfulness and coaching can be useful. In particular, mindfulness can enhance the quality of cognitive and metacognitive performance [27,28,29,30], foster emotional regulation and self-awareness [31], and improve attentive regulation [31,32], and coaching, as defined in a following section, can improve motivational aspects and goal-oriented self-regulation skills fostering the achievement of academic goals [33,34,35,36].

The intervention assessed in this study, called MEL (Mindful Effective Learning) training, aims to improve learning effectiveness and academic achievement in university students by training all three aspects of effective learning: emotional, motivational and self-regulation in study. To achieve this goal, both mindfulness, which works primarily on emotional and attention regulation and on awareness and cognitive performance, and coaching, which primarily works on motivation and goal-oriented self-regulation skills, were used. These two approaches were integrated into a mindfulness-based group coaching that, besides working on emotions and attention regulation, awareness, cognitive performance, motivation and goal-oriented self-regulation skills, focuses also on study strategies and techniques necessary for a self-regulated study [12,13,14,15], as described in the method section.

### 1.2. Mindfulness

Mindfulness meditation was originally practiced in Eastern countries following the Buddhist philosophy. Its diffusion in Western countries become considerable at the end of the 1970s due to the secular contribution of Jon Kabat-Zinn. He proposed the well-known MBSR (Mindfulness-Based Stress Reduction) protocol in 1979 in a clinical context to offer patients a path of mindfulness meditation to treat chronic pain and stress [37]. Due to the benefits initially shown by the mindfulness-based protocol and later assessed by the Western psychology and neuroscience scholars, the Kabat-Zinn protocol spread to the Western countries also in non-clinical contexts like the educational one. Mindfulness can be defined as “a receptive attention to and awareness of present moment events and experience” [38] (p. 212) or “the awareness that emerges through paying attention on purpose, in the present moment, and non-judgmentally to the unfolding of experience moment by moment” [37] (p. 145). The mindfulness meditative practice consists of staying focused on one’s natural breath and physical sensations. Mental formations distract the mind from focusing and it is then necessary first to become aware of it, second to let go without judging or distracting thoughts, and third to come back again to the moment and experience the breathing and body sensations. Those numerous cyclic repetitions train the focused attention on a defined object and the ability to monitorexternal events and mental formations that arise in the present moment with non-judgmental acceptance [32]. It has been shown that focused attention and open monitoring abilities, enhanced by practice, foster emotional regulation and awareness of Self and of the external events [31], as well as quality of cognitive and metacognitive performances [27,28,29,30]. Moreover, it has been shown that mindfulness improves resilience [25,26].

Thanks to its benefits, mindfulness has spread to the West in various areas, including higher education [39,40,41,42,43]. In particular, it has been highlighted that meditative practice improves emotional regulation [31,44,45], attention regulation [31,32], self-awareness [31], and cognitive and metacognitive processes [27,28,29,30] that can be relevant for effective learning and academic achievements [39,40,41,42,43]. It can also be useful to consider Langer’s [46] non-meditative approach to mindfulness, which claims that mindfulness develops flexible thinking, helps to create new conceptual categories, and opens up more prospects for problem solving. Numerous experimental studies have assessed the effectiveness of time-limited (typically 8 weeks) mindfulness-based training offered to university students [47,48,49]. The results have shown that mindfulness improves self-control abilities and awareness (e.g., [50]), increases self-efficacy (e.g., [25,51]), coping strategies (e.g., [52]), that are connected with resilience, enhances attentive skills (e.g., [53]), improves cognitive performance and metacognition (e.g., [54]), and decreases stress, anxiety, and depression (e.g., [47,55,56,57,58]). As a consequence, it is not surprising that mindfulness-based training has been introduced permanently in several universities around the world [40,43,59,60]. 

### 1.3. Coaching

Coaching is a broad term to indicate different activities in different contexts. Coaching used in the intervention can be defined according to the ICF (International Coaching Federation). Coaching, initially proposed by Gallwey [61] in the sport environment and then applied to a corporate context by Whitmore [62], is defined as a personal development methodology based on a partnership between the coach and the client (the coachee) where a thought-provoking and creative process inspires the coachee to maximize his/her personal and professional potential. Coaching is, therefore, a relationship between two partners, the coach and the coachee, in which one of the two partners, the coach, serves the other as a facilitator of awareness. The coach’s facilitating action takes place due to the ability to listen, and then formulate and ask appropriate “powerful” questions to the coachee. The coach supports the coachee in achieving a specific personal or professional goal, provoking creative processes that inspire the coachee to maximize his/her personal and professional potential. It has been shown that the coach can train coachees to move through a “goal-oriented self-regulated cycle”, which consists of a sequence of processes: set a goal, develop a plan of action, perform the action, monitor performance, evaluate performance, and eventually change action [36]. Furthermore, group coaching, in addition to individual coaching, introduces sharing activities that foster insights, understanding and awareness in a safe space where coachees can speak openly [63,64]. Recently, coaching has been introduced in academic educational contexts to improve students’ performance [34], to encourage them to keep attending their university [33] and to positively affect their motivation [35].

### 1.4. Mindfulness-Based Group Coaching

Starting from the benefits that mindfulness and coaching separately give, several authors have introduced mindfulness in the coaching setting in order to enhance their effectiveness [65,66,67,68,69,70]. In particular Cavanagh and Spence [65] emphasize “acceptance” as a factor present in coaching and mindfulness so that the latter can promote the effectiveness of coaching; Collard and Walsh [66] have introduced a mindfulness training in coaching to reduce stress in the coachees and make coaching more effective; Kemp [67] states that coaching and mindfulness are both aspects of education and training and that it might be useful to integrate the two approaches; Passmore and Marianetti [69], first proposed mindfulness for coachees as well as coaches and said that coaching can be improved by a preparatory mindfulness training; finally, Virgili [70] introduced the “Mindfulness-based Coaching” and underlined the importance, in terms of effectiveness, of the attention given to the present moment and of the non-judgmental awareness introduced by the mindfulness in the coaching session.

Inspired by these contributions, the intervention described in this pilot study used a mindfulness-based group coaching approach where, in addition to working on emotions, particularly on anxiety, and resilience, goal-oriented self-regulation skills, self-awareness, meta-cognitive performance and motivation, necessary study strategies and techniques were trained to promote effective learning, as described in the methods section.

Analyzing the literature concerning university training programs to enhance learning effectiveness and academic achievements, numerous contributions have been found based on mindfulness practice (e.g., [40,43,47,49,59]) or coaching (e.g., [34,35]). Moreover, based on the scientific findings regarding study strategies and techniques [12,13,14,15], there are university courses where strategies and techniques are taught as it is possible to find out from the official websites of universities (e.g., [71]). Despite this worldwide opportunity, to our knowledge, no experiences that combine mindfulness, coaching and trainings on study strategies and techniques, oriented to simultaneously acquire self-regulation in study and emotional and motivational abilities have been investigated. Therefore, the mindfulness-based group coaching that includes training on study strategies and techniques used in the MEL intervention might represent an original attempt to provide a holistic short program for university students to improve learning skills and academic achievement. The intervention studied in this paper is based on a holistic approach because learning is a holistic experience. The choice to use coaching, mindfulness and strategies training together aimed to cover as much as possible of all the holistic aspects. This study aimed to investigate the following question: can a short intervention (10 weeks) improve simultaneously the three dimensions of an effective learning, which are self-regulation in study, emotion and motivation? In particular the study assessed the following dimensions before and after the intervention: (1) for self-regulation in study: self-awareness, organization of study activities, elaboration of study content, self-assessment of one’s studying, use of study strategies and techniques, metacognition; (2) for emotions: anxiety regulation; (3) for motivation: theory of own entity/incremental intelligence and personality, confidence in one’s own intelligence and personality, self-ability perception, academic achievement goals; (4) for all the three dimensions: resilience.

## 2. Materials and Methods

### 2.1. Participants

Forty-five students (25 female and 20 male) on the bachelor’s degree course in management engineering at the School of Polytechnic and Basic Sciences at the University of Naples Federico II participated voluntarily in the study. Twenty-four students expressed interest in the MEL training and signed up for the class and were assigned to the intervention group. However, during the intervention, three participants did not attend the minimum 28 h of training, and for this reason, they were not included in the analysis. The intervention group was, therefore, composed of 21 students (11 female and 10 male, 13 from the 1st year and 8 from the 2nd year, mean age M_age_ = 19.86, standard deviation (SD) = 0.94). Prior to attending the university, 18 students studied in scientific high school, two in humanistic high school and one in technical high school; nine of them were off-site students.

The control group was composed of 24 students (14 female and 10 male, 13 from the 1st year and 11 from the 2nd year, M_age_ = 19.75, SD = 0.66). Prior to attending the university, 19 students studied in a scientific high school, four in a humanities high school and one in a technical high school; 10 of the students were off-site students. The control group was selected from students who did not attend MEL training who completed the pre-tests (*N* = 151), balancing for number, gender, and year of university course. The control group completed pre-tests and post-tests but did not participate in the intervention.

**Figure 1:**
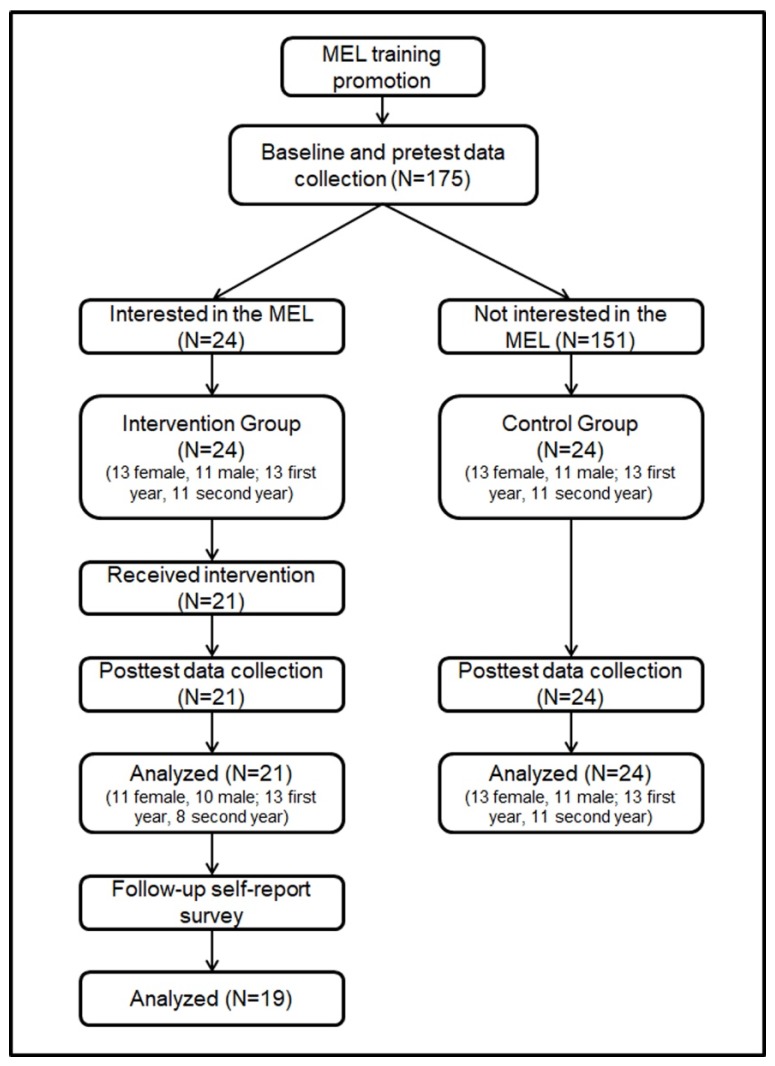
Flow of the study with a pre/post controller design and a follow-up self-report survey.

Students in both the intervention and control groups were Italian and had never practiced meditation before. See Figure 1 for a participant flow diagram. 

### 2.2. Design and Procedure 

A two-group pre-test-post-test design was used to assess the intervention. Participants in both the intervention and control groups were measured two times: (a) before the intervention (pre-test) and (b) after the intervention (post-test). The same questionnaires were used for the pre- and post-test assessments. To increase consistency across the groups, both the intervention and control groups were assessed at the same time, date, and location. The pre-test questionnaires were delivered during the first lesson of the second semester courses. Before submitting the questionnaires, the first author briefly explained the aim of the study and the questionnaires. It was specified that the tests would be anonymous, and the students were informed that the tests would be repeated at the end of the second semester courses.

The questionnaires were anonymous, and it was possible to associate the pre-test and post-test data for a single student by using a nickname.

In addition, one follow-up self-report survey was administered to the trained students six months after the end of the intervention to assess the perceived gains attained through the intervention and another follow-up self-report survey were administered one year after the end of the intervention to assess the academic results achieved after the intervention.

### 2.3. Materials

To measure variables that describe the effectiveness of the intervention, some parts of the AMOS (Abilities and Motivation to Study) questionnaire [72] were chosen for a total of 13 variables: self-regulation in the study (five subscales), anxiety for emotion regulation, motivation (six subscales) and resilience as a transversal ability. The AMOS questionnaire was chosen because it is the only one validated in the Italian university context that investigates the effectiveness of the academic study (Parts of the AMOS questionnaire have been used in the study done by Mega et al. [10] on effective learning and in the study done by Costabile, Cornoldi, De Beni, Manfredi, and Figliuzzi [73] on metacognition.)

In addition, the Italian version of the Mindful Attention Awareness Scale (MAAS) [38,74] was used to evaluate the effect of mindfulness practice on self-awareness. The MAAS was validated in Italy by Veneziani and Voci [74].

### 2.4. Intervention 

The training was called MEL (Mindful Effective Learning) and it was designed as a mindfulness-based group coaching. The MEL lasted 10 weeks, with a total of 10 modules of 3.5 h. The training was conducted by the first author (Regarding coaching skills, she is an associate certified coach (ACC) accredited by the ICF (International Coaching Federation), and regarding mindfulness skills, she is a regular mindfulness meditator, and she has obtained several diplomas for mindfulness trainer. She has been a university professor for about 30 years teaching science subjects, recently she obtained a university degree in communication psychology and wrote a thesis on Self-Regulated Learning.) At the beginning of the 10-week MEL training, participants in the intervention group were split into three groups of 8 students because group coaching is designed for no more than 10 participants. The three intervention classes were trained with the same activities program. 

In the mindfulness-based group coaching used in the MEL training, besides working on emotions and attention regulation, awareness and metacognitive performance and motivation some specific sessions were dedicated to study strategies and techniques. This was carried out in the second part of the training as described below. 

The MEL training consisted in 10 thematic modules. The first module was devoted to creating a comfortable space in the group and introducing the main topics covered in the training. The second and third modules were about basic abilities for effective self-regulated study: self-awareness and attention regulation. The following two modules concerned emotion regulation, in particular, regulating anxiety, and motivation. Then, after working on the aspects of “being”, the following four modules were devoted to the “doing” aspects needed for self-regulated study: study planning and time management, which are related to the ability to efficiently organize one’s study activities; study techniques and mnemonics, which are related to the ability to efficiently elaborate study content, to monitor and evaluate one’s own studying and to use appropriate techniques. The last module focused on the gains from and rewards of the training.

**Figure 2:**
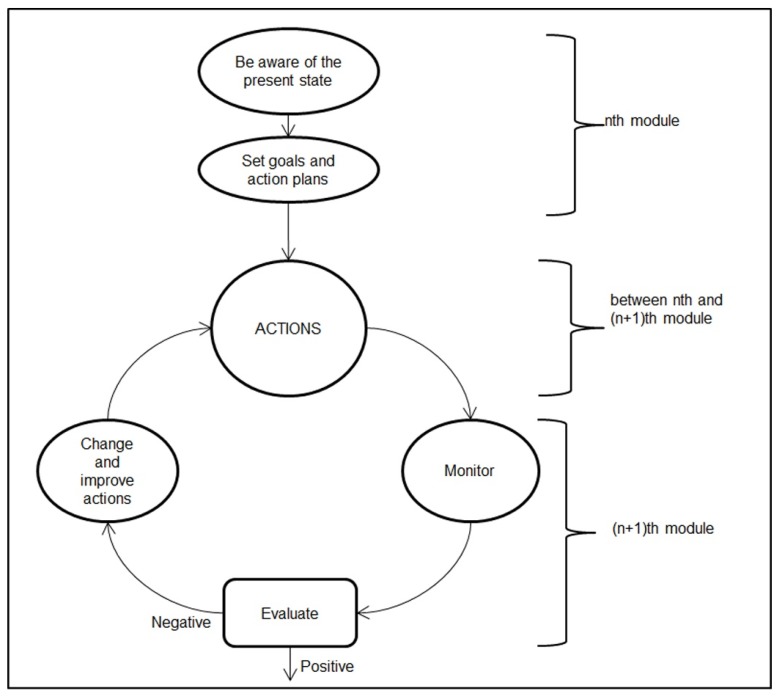
Structure of the training module.

With regard to the structure of the single module of the training, it was designed with the purpose of facilitating students in moving mindfully through the goal-oriented self-regulated cycle [36] shown in Figure 2, in which goals and action plans were designed to help the students learn effectively and to achieve positive academic goals. In particular, in each individual module, the students monitored, evaluated and eventually made improvements and changes to the actions they carried out during the week before the present module, while awareness of the present state, goals and action plans were emphasized for the aspects covered by the present module.

In mindfulness-based group coaching, mindfulness can be introduced in the coaching setting in different ways, as described in Virgili [70]. In the MEL training, this was accomplished through a guided mindfulness meditation at the beginning of each module and just before the specific activities of the group coaching. This practice predisposed the participants to have a mindful attitude in the group coaching activities, which means awareness of the present moment, focused attention and open monitoring while carrying out activities [31,32]. In addition, sometimes a guided meditation practice, oriented to a specific object inherent to the module’s theme, was proposed as an experiential activity in the group coaching session. 

Each module, according to the mindfulness-based group coaching approach, was conducted as follows:

First, the mindfulness meditation practice at the beginning of each module consisted of a formal seated meditation session of up to 30 min, during which the students assumed a comfortable position, closed their eyes, and turned their attention to their own natural breath and the sensations that it generated in the body. 

Second, the coach facilitated group sharing to monitor and evaluate the progress achieved toward goals from the previous week. The coach asked some questions (e.g., “How satisfied do you feel with the study session management you did last week?”), and in turn, the students answered. If the evaluation was negative (e.g., “It was quite good but twice at the beginning of the session I procrastinated for about one hour before starting to study and I couldn’t finish my schedule that day.”), changes and improvements had to be defined for future action (e.g., coach—“What prevented you from starting at the established time?”; student—“I felt anxiety and I was scared to be unable to face the challenge of the study session”; coach—“How can you overcome this obstacle?”; student—“I could meditate for 10 min in order to release and let go of anxiety and fear.”). 

Third, the coach gave some theoretical frameworks to introduce the participants to the module topic: awareness and mindfulness, metacognition, attention regulation, emotions, motivation, study strategies and techniques. Moreover, materials were given for homework in the form of book references, papers, presentations prepared by the coach, videos, and online talks. 

Fourth, group coaching was conducted to develop awareness of the present state and to set goals and action plans regarding the module’s topic, for example, to define personal rules to organize study sessions and define actions to achieve the goal. That effort was carried out with sharing moments and with experiential individual or group activities facilitated by the coach, such as games and role playing, problem solving, trainings on specific techniques, self-report questionnaires, dyadic reciprocal interviews, and guided meditations oriented to a specific object [75]. For the specific activities performed, see Table 1. Guided meditation practice was used before each activity was performed according to the mindfulness-based approach. 

The end was dedicated to sharing the insights gained from the module. 

Specific mindfulness practices moderated by the trainer were used in different occasions during the training. These practices aimed to train the participants on self-regulation skills regarding attention and anxiety, and on awareness of the daily activities and metacognition. In the self-awareness module, the “raisin exercise” from the MBSR protocol [37] consisted in eating a raisin while giving focused attention to all the sensations arising in the body while eating, such as the taste of raisins while chewing in the mouth. This practice allowed participants to improve their awareness of the present moment letting go of “their ideas about what raisins taste like, and to simply “drop in” on the actuality of their lived experience and then to sustain it as best they can moment by moment, with intentional openhearted presence and suspension of judgment and distraction, to whatever degree possible” [37] (p. 148). Another exercise was called “Awareness continuum practice” and it consisted in a dyadic practice where each of the two partners take turns being aware of what arises moment by moment in the mind, such as body sensations, memories, images, thoughts, communicating this stream of consciousness to the partner. This practice trained participants to be mindful moment after moment in the present without judging what arose in the open space of internal awareness. In the attention regulation module, guided meditation using an external object was proposed. In particular, a plant was chosen and put in the center of a participant’s group and they were asked to stay focused on the object for ten minutes observing all the characteristics of it. It was also suggested to welcome the distracting thoughts and gently come back to the main object again and again. Again, guided meditation on an internal object was proposed in this module, presented as a stable image left to arise inside oneself. Both these two practices trained the ability to stay focused on a specific object. In the emotional regulation module: after practicing breath meditation, participants were asked to read a paper with a specific problem-solving task written and to observe the emotions that arose while reading and solving the task. This exercise let the participant become aware of the connection between the anxiety and the task to solve. Moreover, guided meditation based on anxiety release consisted in a body-scan and after in an internal visualization of a squared frame that must travel using the imagination. This exercise trained students to manage anxiety when it arises [55,58]. In the study strategies and techniques module dedicated to the management of time, a task to work on a procrastination tendency [76] consisted of a guided meditation on the emotions that arise when they had to start a study session. This exercise let the participants stay in touch with the discomfort due to the initial moment of the study session and become aware of the procrastination tendency which serves to automatically avoid that discomfort. 

During the sharing time, each group member shared something from his/her own experience, such as emotions, feelings and thoughts; this sharing time was based on so-called active listening, in which those who listen are ready to accept what others bring to the group without judging or interpreting it. The coach must be a facilitator rather than an expert; therefore, he/she does not interfere during the individual sharing but only at the end when, using questions, the coach deepens the members’ self-awareness and facilitates insights and understanding [63]. 

### 2.5. Measures and Data Analysis 

All the participants, before and after the intervention, were asked to complete the following questionnaires in this order:

(1) The MAAS was validated in Italy by Veneziani and Voci [74]. The MAAS was designed to measure the level of awareness of the present-moment experience [38], and it consists of 15 items on a seven-point Likert scale ranging from 1 (almost always) to 7 (almost never) (for example, “I find it difficult to stay focused on what’s happening in the present.”). A total score was obtained by summing and averaging the single items, yielding a total score ranging from 1 to 7. Higher scores indicated higher levels of mindfulness. The Cronbach’s alpha value for this questionnaire was 0.72 at the pre-test and 0.85 at the post-test.
(2) The Study Approach Questionnaire (QAS) is part of the AMOS questionnaire set [72] that investigates self-regulation study abilities. The QAS consists of 50 total items divided into 5 sections of 10 items that cover the following aspects:
  - Organization, that is, the ability to organize and schedule time (for example, “At the beginning of the afternoon, I review all the things I have to do”);
  - Elaboration, that is, the degree of elaboration of the study material (“When I read, I try to formulate questions about the content”);
  - Self-evaluation, that is, the ability to monitor and self-evaluate one’s own learning (“When I have not studied enough, I’m aware of it”);
  - Use of strategies, that is, the ability to use strategies to prepare for an exam (“I try to predict the type of exam/task that awaits me”);
  - Metacognition, that is, the ability to be aware of the functioning of one’s mind (“I like to dwell on thinking about how my mind works”).
    The participants responded to each item using a five-point Likert scale ranging from 1 (never) to 5 (always). The total score was obtained by summing and averaging the single items, yielding a total score ranging from 1 to 5. Higher scores indicated higher levels of efficacy in the study approach. The Cronbach’s alpha values were found to be 0.75 and 0.83 for pre- and post-test organization, respectively; 0.49 and 0.62 for pre- and post-test elaboration; 0.66 and 0.75 for pre- and post-test self-evaluation; 0.69 and 0.67 for pre- and post-test use of strategies; and 0.66 and 0.71 for pre- and post-test metacognition.
(3) The Anxiety and Resilience Questionnaire (QAR) is part of the AMOS questionnaire set [72]. The QAR consists of 14 items, 7 for anxiety (“Anxiety about the exam prevents me from concentrating”) and 7 for resilience (“I overcome agitation and tension, and I recover from the moments of difficulty in study”), on a five-point Likert scale ranging from 1 (do not agree) to 5 (totally agree). A total score was obtained by summing the single items, yielding a total score ranging from 7 to 35. High scores corresponded to high anxiety levels and high resilience levels. The Cronbach’s alpha values were found to be 0.91 for pre- and post-test anxiety and 0.62 and 0.49 for pre- and post-test resilience, respectively.
(4) The Questionnaire on Beliefs (QC) is part of the AMOS Questionnaire [72]. The QC concerns the motivational aspects of academic study. Composed of 29 items, the QC is divided into 6 subscales, with 2 related to incremental theories of Self, 3 to self-confidence, and 1 to academic achievement goals:
  - Incremental theories of Self: theory of one’s own entity/incremental intelligence (8 items, for example, “You have a certain degree of intelligence, and you can do very little to change it”) and theory of one’s own entity/incremental personality (6 items, for example, “Some people have a good personality; others do not, and they cannot change it a lot”). The items employ a six-point Likert scale ranging from 1 (totally agree) to 6 (totally disagree). Item responses for each participant were summed, yielding a total score ranging from 8 to 48 for intelligence and from 6 to 36 for personality. High scores for both aspects corresponded to an incremental theory of self, as defined by Dweck’s Self-theories [20].
  - Self-confidence: confidence in one’s own intelligence (3 items, for example, “Choose between: (a) Usually I think I’m smart/(b) I wonder if I’m smart”). The participant had to choose between the statement (a) or (b) of every item. After that, as regard the chosen statement, he/she had to choose an item from a three-items scale (“not completely true”, “true”, “very true”). To these items it has been attributed a six-point Likert scale ranging from 1 to 6 corresponding to the following choices: 1 point = statement (b) and “very true” from the scale, 2 points = statement (b) and point “true” from the scale, 3 points = statement (b) and “not completely true” from the scale, 4 points = statement (a) and “not completely true” from the scale, 5 points = statement (a) and point “true” from the scale, 6 points = statement (a) and point “very true” from the scale. The item responses for each participant were summed, yielding a total score ranging from 6 to 18. High scores corresponded to good confidence in one’s own intelligence.
    Confidence in one’s own personality (3 items, for example, “(a) Choose between: When I meet new people, (b) I’m not sure I’ll like them/When I meet new people, I’m sure I’ll like them”). The participant had to choose between the statement (a) or (b) of every item. After that, he/she had to choose an item from a three-items scale (“not completely true”, “true”, “very true”). To these items it has been attributed a six-point Likert scale ranging from 1 to 6 corresponding to the following choices: 1 point = statement (a) and “very true” from the scale, 2 points = statement (a) and “true” from the scale, 3 points = statement (a) and “not completely true” from the scale, 4 points = statement (b) and “not completely true” from the scale, 5 points = statement (b) and “true” from the scale, 6 points = statement (b) and “very true” from the scale. The item responses for each participant were summed, yielding a total score ranging from 6 to 18. High scores corresponded to good confidence in one’s own personality.
    Self-ability perception regarding study activities (5 items, for example, “What do you think about: Your study skills?”). The items had a five-point Likert scale ranging from 1 (poor) to 5 (excellent). Item responses for each participant were summed, yielding a total score for the measure ranging from 5 to 25. High scores corresponded to good perception of one’s study skills.
  - Academic achievement goals (4 items, for example, “In a study situation you prefer to face tasks that you already know/new tasks that you have not faced before.”). The participants had to choose one of the two sentences for each item. Each item had a score of 0 or 1. The item responses for each participant were summed, yielding a total score for the measure ranging from 0 to 4. Low scores corresponded to performance goals, and high scores corresponded to mastery goals.
    The following Cronbach’s alpha value emerged: 0.92 for pre- and post-test theory of intelligence; 0.87 and 0.89 for pre- and post-test theory of personality, respectively; 0.82 and 0.77 for pre- and post-test confidence in one’s own intelligence, respectively; 0.78 and 0.67 for pre- and post-test confidence in one’s own personality, respectively; 0.69 and 0.64 for pre- and post-test perception of self-ability, respectively; and 0.86 and 0.75 for pre- and post-test academic achievement goals, respectively.
(5) A follow-up self-report survey was administered to the students in the intervention group six months after the end of the intervention. The students were asked to answer nine open-ended questions regarding their opinions about the training (see Section 3.7 for the questions). Another follow-up self-report survey was administered one year after the end of the intervention to analyze the average grade achieved by the participant. The surveys were anonymously filled out using SurveyMonkey software.

## 3. Results

### 3.1. Preliminary Analyses

T-tests were carried out to verify whether the intervention and control group were similar before the intervention concerning all the variables investigated. The results showed that there were no significant differences between the groups for any of the variables (Table 2), except for organization (M_intervention group_ = 3.52, M_control group_ = 3.97) and self-ability perception (M_intervention group_ = 17.67, M_control group_ = 19.71), in which the mean of the control group was higher than those of the experimental group (Table 2).

### 3.2. Post-Treatment Analyses

To examine the effectiveness of the intervention on self-regulated study, emotion regulation, and motivation, analysis of covariance ANCOVAs and MANCOVAs were carried out with the group (intervention and control) as an independent variable and the post-test scores of each questionnaire as the dependent variables; to take into account differences at the baseline, the post-treatment analyses were performed with the pre-test values as covariates. To protect against Type I errors, Bonferroni correction was applied to all the MANCOVAs performed in the study [77]. 

### 3.3. Self-Awareness of the Present-Moment Experience

The ANCOVA showed that the intervention group score was significantly higher than the control group score, which indicates an increase in mindfulness in the present moment for the intervention group (Table 3). 

### 3.4. Self-Regulated Study

Regarding organization, elaboration, self-evaluation, use of strategies, and metacognition, the multivariate test of the MANCOVA revealed a significant effect for the intervention group: *F* (5,34) = 8.73, *p* < 0.001, *η*^2^_*p*_ = 0.56. Table 4 presents the significant effects that emerged from the univariate tests (Bonferroni correction: *p* < 0.01). The intervention group scores were significantly higher than the control group scores for organization, elaboration, self-evaluation and metacognition. 

### 3.5. Emotional Regulation

From the ANCOVA on the anxiety score, the intervention group score was significantly lower than the control group score (Table 5). 

The ANCOVA on the resilience score revealed no significant difference between the two groups.

### 3.6. Motivation

Concerning the theory of one’s own entity/incremental intelligence and the theory of one’s own entity/incremental personality, the multivariate test of the MANCOVA showed a significant effect for the intervention group: *F* (2,40) = 11.40, *p* < 0.001, *η*^2^*_p_* = 0.36. Table 6 presents the significant effects that emerged from the univariate tests (Bonferroni correction: *p* < 0.025). The intervention group scores were significantly higher than the control group scores for theory of one’s own intelligence and theory of one’s own personality. 

Regarding confidence in one’s own intelligence, confidence in one’s own personality, and self-ability perception regarding study activities, the multivariate test of the MANCOVA revealed a significant effect for the intervention group: *F* (3,38) = 5.25, *p* < 0.01, *η^2^_p_* = 0.29. The univariate tests (Bonferroni correction: *p* < 0.016) showed that the intervention group score was higher than the control group score for confidence in own intelligence (Table 7). 

From the ANCOVA on academic achievement goals, no significant effect emerged. 

### 3.7. Follow-up Self-Report Surveys 

In the first survey (Table 8), the participants stated that the MEL training was useful mainly due to the experiences shared in the group setting and the improvements in awareness and self-regulation abilities. The participants mostly thought that the MEL content was adequate and that more time and meditation practice was needed. The strengths of the MEL were mainly the environment, the sharing and the management of the group; its weaknesses were the limited time, the length of the modules, and the location. Most of the participants suggested increasing the duration of the course and making the modules more distributed and shorter in duration. The participants suggested improving the time organization and the logistics. A total of 79% of the students reported that they benefited from the academic performance training, as confirmed by the later survey on academic results achieved. 

In the second survey, the participants reported the average grade achieved before the MEL training and after its end. The measure of the achieved average grade has been made after one year from the intervention. Results showed that trained students increased their average grades, from 24.65/30 before the intervention to 25.56/30 after one year from the intervention. 

## 4. Discussion 

Although scholars have pointed out how effective learning and positive academic results depend simultaneously on emotional regulation, motivation, and self-regulation in study [2,9,10], to our knowledge, there are no short university interventions that operate on all three of these aspects at the same time. The intervention assessed in this pilot study, called MEL, aimed to overcome this absence, enhancing learning effectiveness among university students by focusing simultaneously on self-regulation in study [1,3], emotion regulation [4,5,6], and motivation [3,8,11]. 

To achieve the purpose of the MEL training, a mindfulness-based group coaching [65,66,67,68,69,70] with specific training based on study strategies and techniques [12,13,14,15] was used. The assumption of the study was that specific variables, characterizing three facets of effective learning, could be improved by the MEL training. Results showed that nine variables out of 14 significantly increased after the intervention.

Concerning self-awareness, which is crucial in self-regulation study activities [2,16,17,19], trained students increased their awareness of the present-moment experience. The score increases observed after the intervention are comparable to the results of a similar study (e.g., [50]) and indicate a good level of awareness [74]. 

Regarding self-regulated study, trained students increased their organizational and elaborative ability to manage study materials, their self-evaluation ability and their metacognition. This is an important result since effective self-regulated learning and good academic results strongly depend on these abilities as widely studied in the literature [1,2,3,11]. Concerning the use of strategies for preparing for an exam, no significant effects emerged between the two groups at the post-test. That result might be because the study strategies for preparing an exam acquired during the school experience are rather stable at the university level and are, therefore, not easily subject to change. Perhaps more activities during the intervention are needed to acquire new strategies.

In relation to the emotional regulation, MEL training seemed to reduce anxiety levels in the experimental group participants; this is an important result since anxiety could hinder learning and, consequently, the achievement of good academic results [5,6,55,56,57,58]. As regard resilience, no significant effect was observed between the two groups post-test. This result could be because mindfulness practice initially nurtures the acceptance of rising emotions, such as frustration, rather than strengthening coping behavior, which requires longer meditative practice [78]. 

Regards the motivational aspects, trained students improved their theories of their own intelligence and personality, approaching an incremental theory of Self, which is firmly related to effective self-regulated learning [3,8,11]. Additionally, the students’ confidence in their own intelligence significantly improved and this is an important result since effective learning and good academic results depend on positive self-perception and self-efficacy [7,23,24]. However, no significant difference emerged between the two groups for confidence in one’s own personality. Additionally, no difference between the two groups emerged for self-ability perception regarding study activities. It is possible that self-ability perception regarding study activities requires more time to change and more meditative practice, as underlined by Sedlmeier and colleagues [78]. Finally, regarding the academic achievement goal, no significant effect was found between the two groups at the end of the intervention. Certainly, in future training, more attention should be paid to achievement goals. 

Consistent with the above positive results, trained students in the follow-up self-report survey positively assessed the MEL training and reported experiencing academic performance benefits. As regards academic performance benefits, conducting follow-up objective measures in future research, analyzed statistically and compared to an opportune control group, could be desirable.

Another consideration can be done in regard to the duration of the intervention. The MEL training consisted of 10 modules and lasted 10 weeks in all. Its short duration made the proposed training sustainable for students involved in a demanding university course such as the management engineering course.

Some limitations should be underlined when interpreting the results of this study. 

One limitation is related to the intervention design and in particular on the composition of the groups. First, the number of participants was small because the intervention was designed for groups of up to 10 people and provided to manage only three groups. Concerning the small numerosity of the intervention group, in a recent systematic review of randomized and non-randomized controlled trials in the health professional context, McConville and colleagues [47], have assessed a sample of 19 mindfulness-based interventions among 5355 candidates and half of them concerned intervention groups of thirty or less participants. This is due to the fact that some mindfulness-based interventions, such as MBSR, include a small number of participants. This is a pilot study and it is desirable that, in the future, other studies can be carried out with a larger number of participants. Second, the experimental group was self-selected and included students who chose to attend the training. Therefore, the composition was not based on random assignment. For this reason, we balanced the two groups according to number, year in the course and gender, selecting the control group from the students who did not attend the training. T-tests at the baseline showed that the groups were similar at the pre-test for all the measures, except for organization and self-ability perception in which, however, the scores of the control group were higher. Moreover, in order to control for any differences between the two groups before the intervention, post-test scores were obtained by controlling for the pre-test scores. It is understandable that the self-selection of the experimental group, on a voluntary basis, is a limit of the study, but it is the most commonly used selection method for mindfulness-based interventions in university contexts [47]. Still, with regard to the intervention design, due to the promising results achieved in this pilot study it will be interesting to carry out a future study to see the comparison between the control group, the experimental group and another experimental group with another kind of self-regulation training (randomly assigning students who want to participate to one of the experimental groups). Furthermore, it can be noted that in the present study it is not possible to understand how each outcome depends on the single approach of the intervention (mindfulness, coaching, study strategies training). The aspects of effective learning (emotions, motivation, self-regulation in study) are contiguous and it is difficult to discriminate exactly which approach is favored. For example, self-regulation in study, which consists of the attention, emotional and the goal-oriented aspects, is promoted by the three approaches simultaneously. In future studies, it would be desirable to design an intervention which aims to assess how each aspect depends on the single approach; it might be interesting to compare the group treated with the MEL intervention with the groups only treated with certain approaches of MEL (for example, mindfulness and strategy training, or mindfulness and coaching) to verify what changes can be observed in the results.

Another limitation is that the coach conducted the assessment of the intervention and was a professor of regular courses at the university. As a consequence, the students’ answers could be influenced by social desirability bias. In any case, each survey was anonymous, so the identity of the student was hidden, and this modality probably helped the students to feel free to express their opinions. For example, 83% of the students reported some weaknesses of the training in the follow-up survey. Moreover, although 22% of the participants indicated an appreciation for the coach, the same percentage of students expressed satisfaction with the course for other reasons such as the content and the group sharing aspect. 

With regard to the chosen assessed variables, this study did not measure some important variables involved in effective self-regulated learning, such as attention abilities [54] and positive academic emotions [6]. In future studies, it might be possible to optimize the questionnaires and introduce more assessment variables in order to achieve a clearer vision of the effects of the training. Moreover, with regard to the significant results, it is evident that some Cronbach’s alphas are low, but the values obtained are similar to the ones reported in manual of the AMOS questionnaire [72], which are the only ones validated in the Italian context.

Lastly, the generalization of the results of the present study is compromised by the fact that the trained students come only from an engineering academic school (School of Polytechnic and basic Sciences at the University of Naples Federico II). The present work is only a pilot study and in future research it will be interesting to test these concepts within different academic contexts. 

## 5. Conclusions

The short 10-week intervention assessed in this pilot study, called MEL, designed as mindfulness-based group coaching [65,66,67,68,69,70] with specific training based on study strategies and techniques [12,13,14,15], aimed at improving learning effectiveness among university students. According to the existing literature [2,9,10], the starting point of this paper was that the learning effectiveness depends simultaneously on metacognitive and affective skills in addition to cognitive and academic knowledge and skills that can be classified in the following three aspects: self-regulation in study [1,2], emotion regulation [4,5,6], and motivation [3,8,11]. In order to assess the effectiveness of the MEL training regarding its aim, specific variables, characterizing the mentioned three facets of effective learning, have been assessed. Results showed that nine variables out of 14 significantly increased after the intervention. In particular the results showed that, regarding self-regulation in study, trained students improvedtheir self-awareness, self-evaluation ability, metacognition skills, and organizational and elaborative ability to manage study materials; regarding emotional aspects, they improved their anxiety control; regarding motivation, they developed an incremental theory of Self and improved their confidence in their own intelligence. 

In conclusion, according to the results, the MEL training seems to improve students’ effective self-regulated learning. The ability to effectively self-regulate their own study is crucial for university students who must autonomously and completely manage their academic careers through planning and organization. Learning effectively helps students achieve better results and, subsequently, reduces the number of university drop-outs. Furthermore, the MEL training might give students the opportunity to attain personal growth during the university experience, which can sometimes prove a frustrating experience. Despite the limitations of the present pilot study highlighted in the discussion, the results seem to be promising and encourage new research. 

## Figures and Tables

**Table 1:** Activities involved in the mindfulness-based group coaching for each module.

Modules		Activities
1	Introduction	Sharing personal information and expectations.
		Ice breaker game to create a comfortable space in the group. 10 min of meditation.
2	Awareness and metacognition	Raisin exercise from the MBSR protocol * Sharing insights in the group.
		Training on metacognition: recognize all the cognitive activities that arose while reading and understanding a text. Sharing insights in the group.
		Awareness continuum practice **
3	Attention regulation	20 min of guided meditation using an external object: a cloth hanger put in the center of the room. Sharing the experience with the group.
		20 min of guided meditation on an internal object: a stable image arises inside oneself. Sharing the experience with the group. 10 min of meditation.
4	Emotion regulation	Guided meditation on emotions.
		Self-report questionnaire to map one’s own academic emotions. Sharing insights in the group. Training on emotion regulation: awareness of the anxiety that arose during a proposed problem solving.
		Guided meditation on anxiety release.
5	Motivation	20 min of meditation. Theories of Self: dyadic reciprocal interview and sharing in the group on what emerged.
		Self-perception—role playing: group of three people taking turns in the roles “I am”, “I should be”, “I wish to be”. Sharing the insights.
		Self-report questionnaire to become aware of actual academic goals. Sharing in group to set new goals. 10 min of meditation.
6	Study strategy and techniques: Planning to study	30 min of meditation. Individual work to draft a personal study plan, taking into account exams to be completed, time available, and study load. 10 min of meditation.
7	Study strategy and techniques: Managing time	Training on the procrastination tendency: guided meditation on the emotions that arise when we have to start a study session.
		Training on the cycle closure ***: dyadic reciprocal interview to define which cycles are open in the present moment and how to close or delay them.
		Self-report questionnaire and individual work to define the personal rules that best fit the individual study session. Sharing in the group. 10 min of meditation.
8	Study strategy and techniques: Techniques	20 min of meditation. Game on the different techniques: participant chooses a particular technique among interleaving practice, spaced retrieval, self-testing ****, group study, and organizing materials. Students reflect on it and then give a short presentation on the technique. 10 min of meditation.
9	Study strategy and techniques: Mnemonics	20 min of meditation. Game on different mnemo-techniques: split the group into four dyads, each of them uses a different mnemo-techniques; the coach reads a list of words that the students memorize; the efficacy of the different techniques is compared. 10 min of meditation.
10	Conclusion	10 min of meditation. Students presentations on the insights and benefits of the MEL (Mindful Effective Learning) course.

* MBSR: Mindfulness-based stress reduction protocol [37]. ** Awareness continuum practice: awareness and communication of what arises moment by moment in our mind, such as body sensations and thoughts. *** Cycle closure: the act of closing a life experience. **** Effective study strategy and techniques [12].

**Table 2:** Preliminary analysis.

Assessed Variables	*t* (43)	*p*
Self-awareness	−0.89	0.38
Organization	2.84	0.007
Elaboration	0.68	0.50
Self-assessment	−0.05	0.96
Use of strategies	1.5	0.14
Metacognition	−0.53	0.60
Anxiety	−0.44	0.66
Resilience	−0.15	0.88
Theory of own entity/incremental intelligence	1.54	0.12
Theory of own entity/incremental personality	2.06	0.05
Confidence in own intelligence	0.13	0.90
Confidence in own personality	−1.9	0.06
Self-ability perception	2.55	0.01
Academic achievement goals	0.59	0.56

**Table 3:** Self-awareness of the present-moment experience (Mindful Attention Awareness Scale, MAAS).

Self-Awareness	Intervention Group			Control Group		*F* (1,42)	*p*	*η^2^_p_*
	Pre-Test	Post-Test	Pre-Test		Post-Test			
	M (SD)	M (SD)	M (SD)		M (SD)			
Self-awareness	4.85 (0.77)	5.30 (0.84)	4.66 (0.66)		4.60 (0.79)	8.17	<0.01	0.16

**Table 4:** Learning and studying abilities (Study Approach Questionnaire, QAS).

Self-Regulated Study Title	Intervention Group		Control Group		*F* (1,38)	*p*	*η^2^_p_*
	Pre-Test	Post-Test	Pre-Test	Post-Test			
	M (SD)	M (SD)	M (SD)	M (SD)			
Organization	3.52 (0.61)	3.94 (0.75)	3.97 (0.42)	3.84 (0.52)	13.0	<0.01	0.25
Elaboration	3.58 (0.44)	3.85 (0.45)	3.67 (0.35)	3.65 (0.41)	8.98	<0.01	0.19
Self-evaluation	3.72 (0.59)	4.08 (0.49)	3.71 (0.26)	3.60 (0.37)	29.38	<0.001	0.44
Metacognition	3.68 (0.59)	3.99 (0.38)	3.59 (0.48)	3.47 (0.49)	18.93	<0.001	0.33

**Table 5:** Anxiety (Anxiety and Resilience Questionnaire, QAR).

Emotional Regulation Title	Intervention Group		Control Group		*F* (1,42)	*p*	*η^2^_p_*
	Pre-Test	Post-Test	Pre-Test	Post-Test			
	M (SD)	M (SD)	M (SD)	M (SD)			
Anxiety	20.48 (7.96)	15.81 (6.19)	19.54 (6.39)	19.04 (6.92)	7.78	<0.01	0.16

**Table 6:** Theory of own intelligence and personality (Questionnaire on Beliefs, QC).

Theory of Intelligence/Personality Title	Intervention Group		Control Group		*F* (1,41)	*p*	*η^2^_p_*
	Pre-Test	Post-Test	Pre-Test	Post-Test			
	M (SD)	M (SD)	M (SD)	M (SD)			
Theory of own entity/incremental intelligence	28.33 (10.89)	37.43 (11.66)	32.71 (7.57)	34.58 (6.45)	6.96	=0.012	0.14
Theory of own entity/incremental personality	18.00 (6.88)	26.14 (7.33)	21.96 (6.02)	20.75 (6.45)	22.17	<0.001	0.35

**Table 7:** Confidence in own intelligence (QC questionnaire).

Self-Confidence Title	Intervention Group		Control Group		*F* (1,40)	*p*	*η^2^_p_*
	Pre-Test	Post-Test	Pre-Test	Post-Test			
	M (SD)	M (SD)	M (SD)	M (SD)			
Confidence in own intelligence	11.05 (4.82)	14.48 (3.09)	11.21 (3.74)	11.42 (3.28)	15.51	<0.001	0.28

**Table 8:** Follow-up self-report survey.

Questions	Answer	%
Do you think the MEL training was useful?	Yes	100%
	No	0%
If you think it was useful, what was particularly helpful for you?	Group sharing	32%
	Awareness and self-regulation improvement	26%
	Awareness improvement	21%
	Self-regulation improvement	21%
Regarding the contents of the training, would you remove or add something?	No	37%
	Not remove, but deepen some content	26%
	Not remove, but add more meditation practice	21%
	Not remove, but add more time	16%
What are the strengths of the training?	The ambience and the group sharing	45%
	The coach	22%
	The content	22%
	The focus on personal development	11%
What are the weaknesses of the training?	The lack of total time and the length of the modules	50%
	The location	22%
	There are none	17%
	Too little practice	11%
What would you improve?	I would add more time	37%
	I would improve the organization	32%
	I would deepen some content	16%
	None	16%
Do you think the MEL training has improved your academic performance?	Yes	79%
	No	21%
Is the overall duration of the training adequate?	Yes	53%
	No	47%
	No because … I would expect more time for deepening the content	78%
	No because … I would hold more meetings of lesser duration	22%
Is the duration of the single module adequate?	Yes	68%
	No	32%
	No because … I would split the modules	67%
	No because … I would schedule every module duration according to the content	33%
